# Development of a human phage display-derived anti-PD-1 scFv antibody: an attractive tool for immune checkpoint therapy

**DOI:** 10.1186/s12896-022-00752-8

**Published:** 2022-08-23

**Authors:** Sepideh Safaei Ghaderi, Farhad Riazi-Rad, Elmira Safaie Qamsari, Salman Bagheri, Fatemeh Rahimi-Jamnani, Zahra Sharifzadeh

**Affiliations:** 1 Department of Biotechnology, Faculty of Advanced Science and Technology, Tehran Medical Sciences, Islamic Azad University, Tehran, Iran; 2 Department of Immunology, Pasteur Institute of Iran, Tehran, Iran; 3 Immunology Research Center, Tabriz University of Medical Sciences, Tabriz, Iran; 4 Department of Immunology, Tabriz University of Medical Sciences, Tabriz, Iran; 5 Department of Mycobacteriology and Pulmonary Research, Pasteur Institute of Iran, Tehran, Iran; 6 Microbiology Research Center, Pasteur Institute of Iran, Tehran, Iran

**Keywords:** PD-1, Single-chain fragment antibody, Immune checkpoint inhibitor, Immunotherapy

## Abstract

**Background:**

The PD-1 checkpoint pathway plays a major role in tumor immune evasion and the development of the tumor microenvironment. Clinical studies show that therapeutic antibodies blocking the PD-1 pathway can restore anti-tumor or anti-virus immune responses by the reinvigoration of exhausted T cells. Because of the promising results of anti-PD-1 monoclonal antibodies in cancer treatment, autoimmune disorders, and infectious diseases, the PD-1 has emerged as an encouraging target for different diseases.

**Results:**

In the present study, we employed a human semi-synthetic phage library for isolation of some scFvs against the extracellular domain of PD-1 protein by panning process. After the panning, a novel anti-PD-1 scFv (SS107) was found that exhibited specific binding to PD-1 antigen and stimulated Jurkat T cells. The selected anti-PD-1 scFv could restore the production of IL-2 and IFN-γ by Jurkat T cells that were co-cultured with PD-L1 positive tumor cells.

**Conclusion:**

This anti-PD-1 scFv with high specificity and the ability to reactivate exhausted T cells has the potential to be developed as an anti-cancer agent or to be used in combination with other therapeutic approaches.

**Supplementary Information:**

The online version contains supplementary material available at 10.1186/s12896-022-00752-8.

## Background

PD-1 or programmed death 1 is a critical inhibitory member of the CD28 receptor family that was originally isolated and characterized from T cell hybridoma by subtractive hybridization technique in 1992 [1]. PD-1 expression has been found on B cells, activated T cells, natural killer cells, dendritic cells, and monocytes. PD-1 is a 50–55 kDa (288 amino acid) type I transmembrane protein of the immune globulin superfamily, containing an extracellular single IgV domain, a transmembrane domain, and a cytoplasmic tail [1, 2]. The cytoplasmic tail region of PD-1 involves an ITIM (immuno-receptor tyrosine-based inhibitory motif) and an ITSM (immuno-receptor tyrosine-based switch motif), and the last one is vital for the down-regulation and inhibitory function of TCR signaling [3]. The PD-1 checkpoint pathway in peripheral tissues acts as a negative regulatory checkpoint molecule to dampen overstimulation of immune responses and leads to keeping of immune tolerance to prevent the destruction of self-tissues [4]. PD-1 receptor has two known B7 family ligands: PD-L1 (B7- H1) [5] and PD-L2 (B7-DC) [6], which have different expression mechanisms on cells. PD-L1, the principal mediator of immunosuppression, is expressed by many human tumors, consisting of kidney, lung, and melanoma [5, 7, 8]. PD-L2 expression is limited only to dendritic cells and macrophages and in some human tumors [9]. Binding of PD-1 to B7 family ligands PD-L1 and PD-L2 leads to delivering inhibitory signals into activated T cells, suppressing effector T cell function and proliferation, inducing apoptosis/ deletion, down-regulating pro-inflammatory cytokines, and shutting down the anti-tumor immune responses [10]. The ability of tumor cells to express PD-L1 is one way whereby tumors exploit to evade immune attack [11]. Different autoimmune disorders can develop in PD-1-deficient mice, including myocarditis and lupus-like autoimmune diseases [12, 13]. Blocking of the PD-1/PD-L1 interaction promotes anti-tumor activity in several syngeneic mouse models [14]. Several data have demonstrated that blocking of PD-L1 and PD-L2 by antagonist antibodies led to an augmented proliferation of functional human and murine antigen-specific T cells [15, 16]. Moreover, another study showed that augmentation of PD-1 expression on CD4+ and CD8+ T cells of gastric cancer patients was notably higher than that of normal controls, which demonstrated the increased PD-1 expression was related to the immune evasion of tumor cells [17]. Based on this important evidence, the PD-1 pathway has appeared as another encouraging target for cancer therapy. Hence, antibodies that block the PD-1 pathway can restore anti-tumor immune responses and promote tumor regression in animals [18]. Previous studies showed that PD-1 blockade led to the augmentation of effector T cells in tumor sites, increasing the cytolytic activity of tumor-specific cells, increasing production of IL-2, INF-γ, TNF-α, IL-17, and IL-6, and decreasing the production of the Th2 cytokines such as IL-5 and IL-13 [19, 20]. Furthermore, different therapeutic strategies such as antagonism of receptor/ ligands interactions by whole monoclonal antibodies (mAb) and combination therapies have been investigated in clinical trials on several types of malignancies [21]. Monoclonal antibodies and their derivatives represent valuable tools in biopharmaceuticals fields but the immunogenicity of hybridoma-derived antibodies and nonspecific binding of the Fc domains of mAbs lead to impaired therapeutic applications [22]. The small antibody fragments possess several advantages over full-length mAbs including ease of production, faster tissue penetration, less immunogenicity and the lack of Fc-mediated activation of the immune system. Phage display technology has broadly been used for selection of recombinant antibody fragments. This technique makes it easy to select specific binders against various target molecules [23, 24].

The smaller antibody fragments, such as single-chain variable fragments (scFv) and antigen-binding fragments (Fab), have been developed by phage display technology [25, 26]. An scFv is an engineered antibody fragment including variable light (VL) and heavy (VH) chains of an antibody that are linked by a flexible polypeptide linker, thereby forming the antigen-binding domain [27]. These antibody fragments have several benefits compared to the whole mAbs, including smaller size, superior tissue-penetrating into tumor tissues, and large-scale expression in the *Escherichia coli* (*E. coli*) system. For these reasons, scFvs are known as valuable tools for many diagnostic and therapeutic purposes [28].

In this project, we used phage display technology to isolate scFvs from a human semi-synthetic phage library (Tomlinson I and J) against the extracellular domain of human PD-1 protein. For this goal, after four rounds of antigen panning with human PD-1, specific anti-PD-1 scFv-phage particles were isolated and utilized for more analyses. Selected scFvs were characterized by enzyme-linked immunosorbent assay (ELISA), western blotting, and sequencing. Furthermore, the functionality of anti-PD-1 scFvs was assessed through analysis of cell surface binding that was detected by flow cytometry. Expression levels of IL-2 and INF-γ in the culture supernatants were measured by ELISA.

## Results

### Selection of specific PD-1 scFvs

Human single-chain scFv libraries I + J (Tomlinson I + J) were amplified and employed to select scFvs against PD-1 protein. The phage titers of four outputs were estimated by counting the colony-forming units (CFUs) of the infected *E. coli* TG1. After four rounds of panning, the phage titer of outputs was increased from 10^4^ (first round) to 10^8^ (fourth round) CFUs/ml (Table 1). The enrichment factor was determined by calculating the ratio between the input number of phage particles at the beginning of each experiment and those recovered at the end. As shown in Table 1, the enrichment factor considerably increased from 3 to 15,662, indicating the enrichment of PD-1-specific phages.

**Table 1:** The titer of outputs and inputs from four rounds of biopanning

Panning round	Tween 20 (%)	Input phages^a^	Output phages^a^	Ratio	Enrichment factor^b^
1	0.5	1.2 × 10^12^	2 × 10^4^	8.3 × 10^–9^	1
2	1	3 × 10^12^	8 × 10^4^	2.6 × 10^–8^	3
3	2	1.1 × 10^12^	3 × 10^6^	1.8 × 10^–6^	216
4	4	2.3 × 10^12^	8 × 10^8^	1.3 × 10^–4^	15,662

^a^The phage titers of inputs and outputs were estimated by counting the colony-forming units (CFUs) of the infected *E. coli* TG1

^b^Enrichment factor was calculated by dividing the output/input ratio in each round of biopanning by the ratio in the first round

### Polyclonal and monoclonal phage screening

**Fig. 1:**
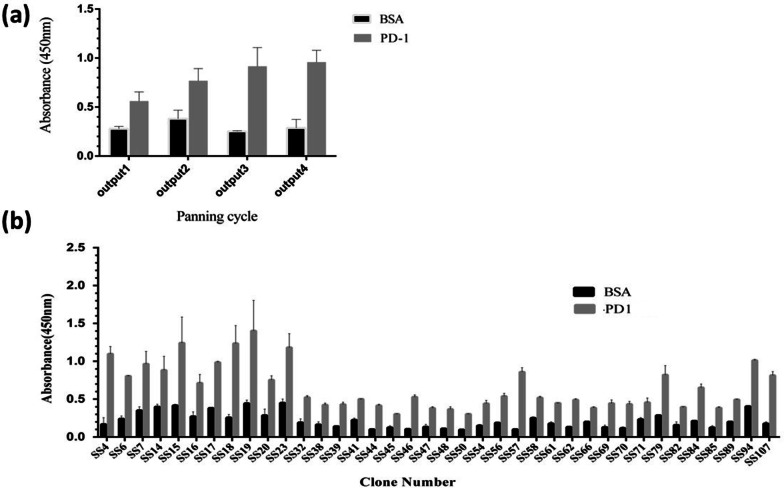
**a** Polyclonal and **b** Monoclonal Phage ELISA of selected scFvs against PD-1protein. Data are shown as means [± SD] of OD values from triplicate experiments. An arbitrary cutoff three times greater than the negative control (BSA-coated wells) was considered positive

Eluted phage clones (outputs) from rounds 1, 2, 3, and 4 were evaluated for specific PD-1 binding properties. As shown in Fig. 1a, the optical density (OD) values of wells coated with PD-1 protein were higher than the bovine serum albumin (BSA)-coated wells (control) and increased round by round, indicating the recovery efficiencies of the specific anti-PD-1 phage clones in the phage display biopanning. Then, around 120 randomly single phage clones from the third and fourth rounds of panning were picked, expanded, and tested for specific target binding in monoclonal phage ELISA. Thirty-eight distinct clones represented positive reactivity to PD-1 protein and were selected for further analysis (Fig. 1b).

### Soluble scFv screening and DNA sequencing

**Fig. 2:**
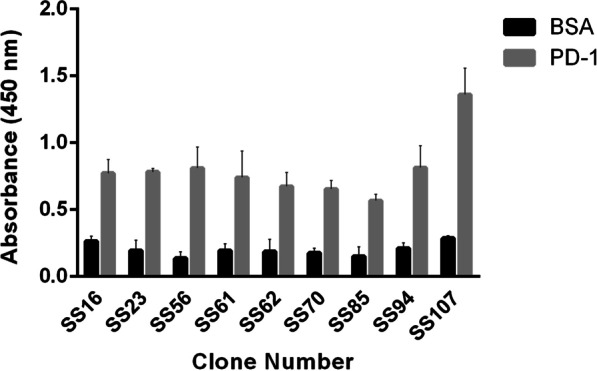
Binding of soluble monoclonal scFvs to PD-1 and BSA-coated (negative control) wells. Data are presented as means [± SD] of OD values from triplicate experiments. An arbitrary cutoff three times greater than the negative control was considered positive

A total of 38 positive anti-PD-1 scFv clones from the monoclonal phage ELISA were employed to infect *E. coli* Rosetta-Gami-2 cells. After IPTG induction and periplasmic extraction, their binding specificity was analyzed by ELISA, and nine soluble scFvs were identified, showing the highest binding with the PD-1 protein (an OD > threefold greater than the OD associated with BSA-coated wells) (Fig. 2).

Following soluble ELISA, the plasmid of nine positive scFv clones was isolated and amplified through PCR with LMB3 and pHEN primers. The amplified phage DNA products were checked for the presence of the desired bands (~ 935 bp) on 1% agarose gel. Four out of nine scFv clones presented a band with the corresponding size (data not shown). Nucleotide sequencing revealed that all four clones had the same sequence. The framework regions (FRs) and complementarity determining regions (CDRs) were determined by the alignment of this sequence in the IMGT/V-Quest software program. This anti-PD-1 scFv (referred to as SS107), whose amino acid sequence is shown in Table 2, was taken for further analysis.

**Table 2:** The deduced amino acid sequence of anti-PD-1 SS107 scFv selected from the human phage library

**SS107/VH**	**FR1**	**CDR1**	**FR2**	**CDR2**
	EVQLLESGGGLVQPGGSLRLSCAAS	GFTFSSYA	MSWVRQAPGKGLEWVSY	ITKAGSNT
	**FR3**	**CDR3**	**FR4**	**LINKER**
	TYADSVKGRFTISRDNSKNTLYLQMNSLRAEDTAVYYC	AKASGPFDY	WGQGTLVTVSS	GGGGSGGGGS GGGGS
	**FR1**	**CDR1**	**FR2**	**CDR2**
**SS107/VL**	DIQMTQSPSSLSASVGDRVTITCRAS	QSISSY	LNWYQQKPGKAPKLLIY	QAS
	**FR3**	**CDR3**	**FR4**	
	TLQSGVPSRFSGSGSGTDFTLTISSLQPEDFATYYC	QQVSGSPVT	FGQGTKVEIK	

The framework regions (FRs) and complementarity determining regions (CDRs) were determined using the IMGT database (www.imgt.org)

### Specificity

**Fig. 3:**
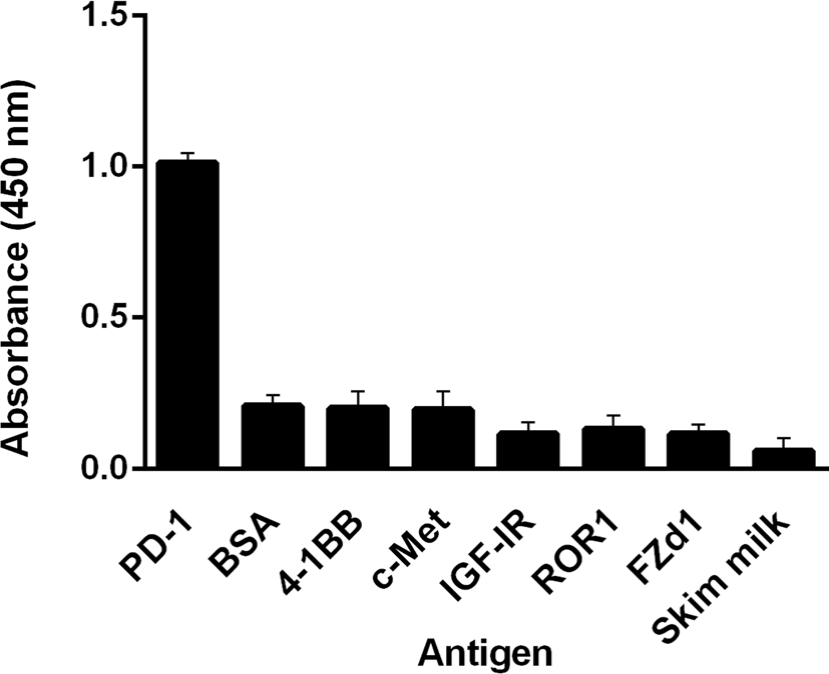
Specificity of the selected anti-PD-1 scFv. The binding specificity of the soluble anti-PD-1 SS107 scFvs to several immobilized peptides and proteins was determined through ELISA. The data are presented as mean ± SD from triplicate experiments. Peptides: c-Met,4-1BB (CD137), IGF-1R, ROR1, Fzd7. proteins: PD-1, skim milk, and BSA

The possible cross-reactivity of anti-PD-1 scFv with other proteins such as 4-1BB, c-Met or HGFR (hepatocyte growth factor receptor), IGF-1R (insulin-like growth factor 1 receptor), ROR1 (receptor tyrosine kinase-like orphan receptor 1), Fzd7 (frizzled-receptor 7), skimmed milk, and BSA was investigated through ELISA. As indicated in Fig. 3, the selected scFv was highly specific for the PD-1 antigen, and no cross-reactivity with other antigens was seen.

### Western blotting

**Fig. 4:**
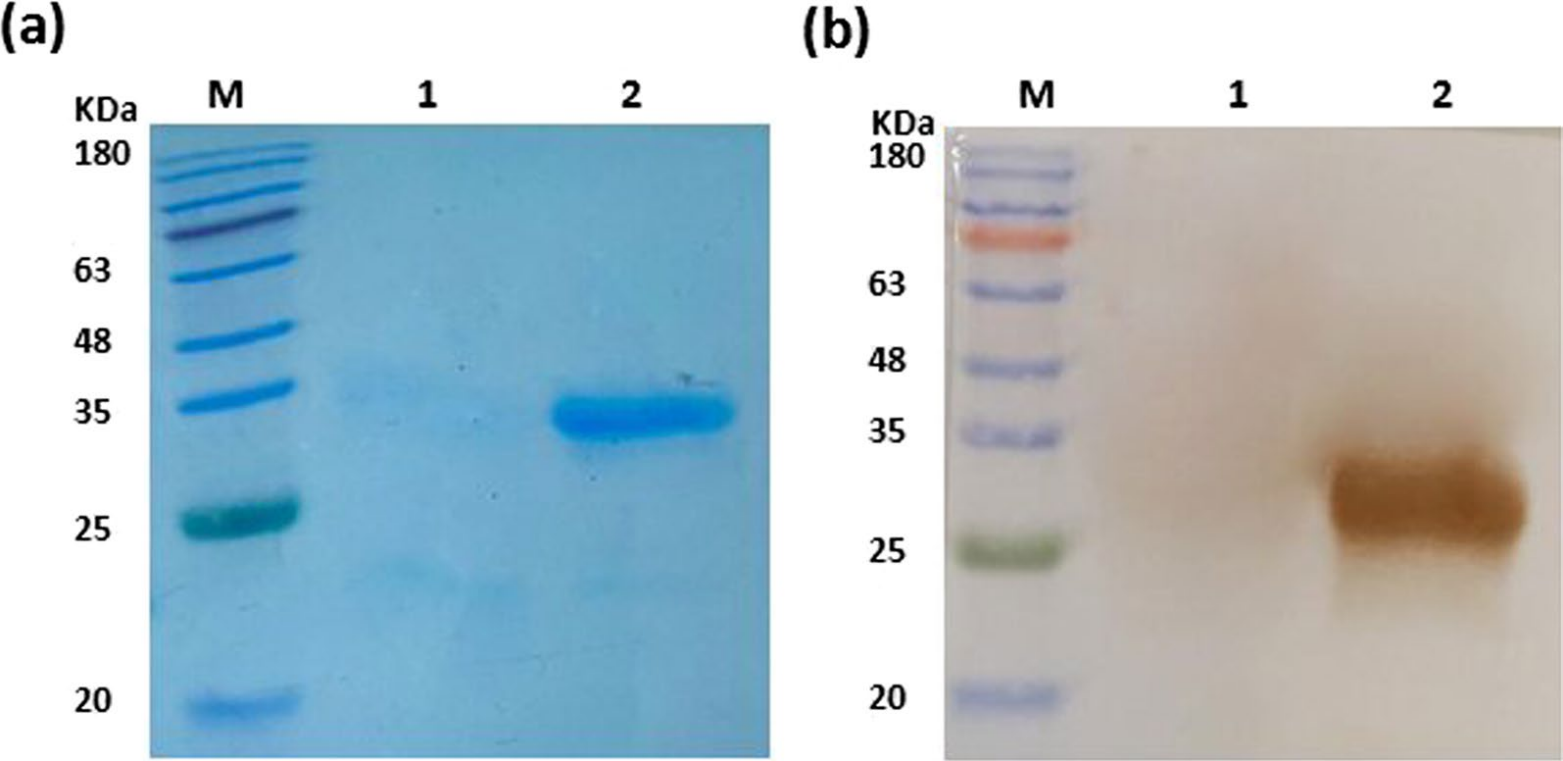
The expression of soluble scFv fragment in Rosetta-Gami 2 was evaluated by SDS–PAGE (**a**) and Western blot analysis (**b**). M: protein marker; Lane 1: total lysate from non-induced *E. coli* Rosetta-Gami 2 as negative control; Lane 2: SS107 scFv; The molecular weight of SS107 scFv was about 28 kDa

Western blot analysis was carried out to examine the expression of soluble SS107 antibody. The results confirmed the presence of anti-PD-1 scFv protein with a molecular weight of ~ 28 kDa (Fig. 4).

### Flow cytometric analysis for cell binding property of SS107 scFv

To show the cell binding ability of SS107 scFv, the stimulated Jurkat T cells were treated with the selected phage-displayed scFv and a commercial mouse anti-PD-1 IgG as a positive control. Unstimulated Jurkat T cells treated with SS107 phgae-displayed scFv and an irrelevant scFv, anti-c-met scFv, were included as negative controls.

**Fig. 5:**
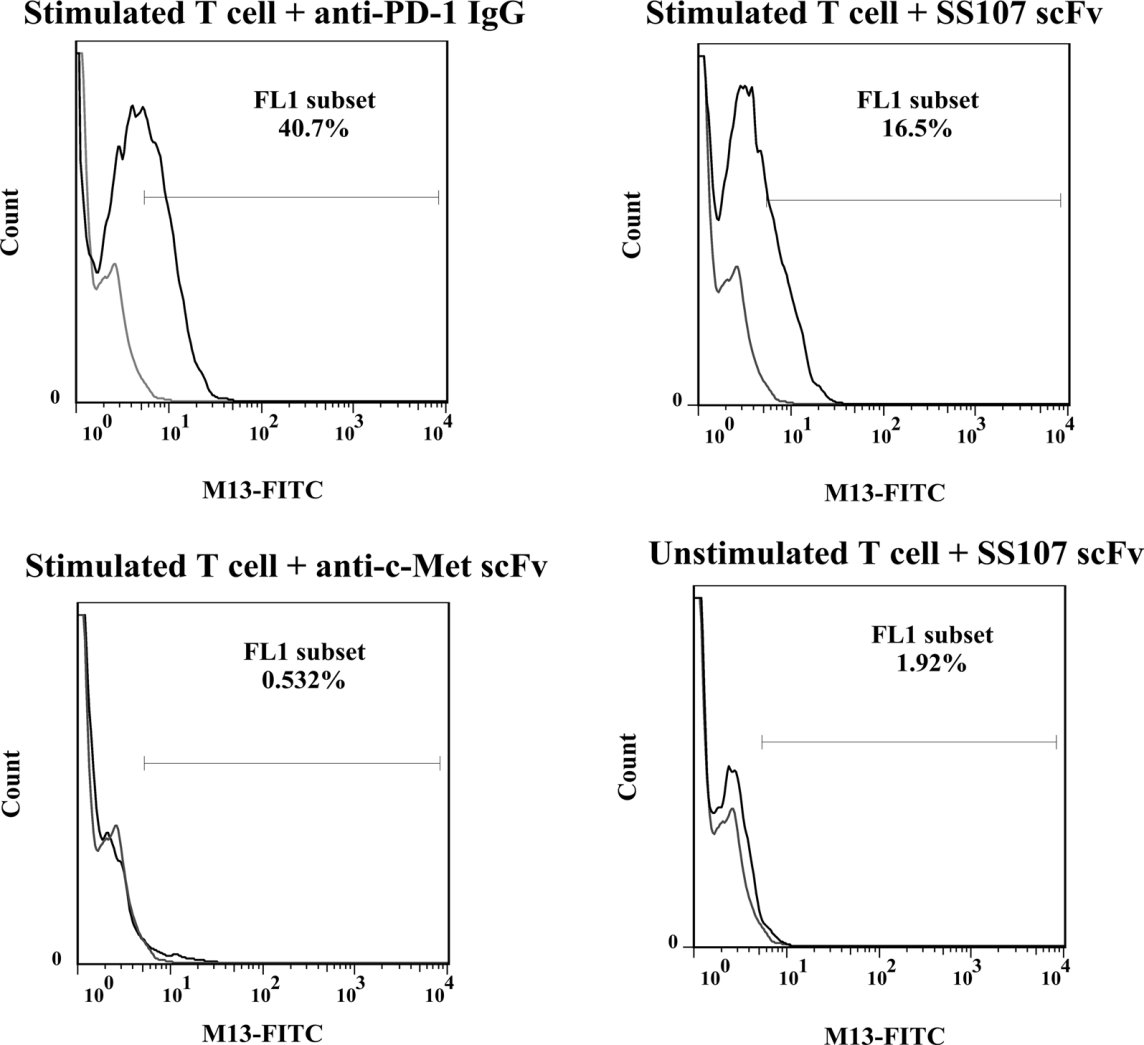
Flow cytometry binding analysis of the selected scFvs. The stimulated Jurkat T cells were treated with the selected scFvs (SS70, SS 107, SS94, and SS85) and a commercial mouse anti-PD-1 mAb as a positive control (C+) and then stained with FITC conjugated anti-M13 for flow cytometry analysis. Unstimulated Jurkat T cells and anti-c-met scFv as an irrelevant scFv were included as negative controls. Thin line: isotype control, bold line: scFv or commercial anti-PD-1 antibody

The living cells were gated according to their FSC and SSC characteristics. Then, the binding of the phage-displayed scFv to Jurkat cells was analysed by anti-M13-FITC antibody. As shown by the shift in fluorescence intensity value of the cells stained by isotype control, commercial anti-PD-1 IgG and SS107 scFv bound to around 40.7% and 16.5% of the stimulated Jurkat cells, respectively. The fluorescence intensities obtained from the unstimulated cells stained with SS107 scFv and the stimulated cells stained with an irrelevant scFv were 1.9% and 0.53%, respectively (Fig. 5).

### Effect of SS107 scFv on human T cell IL-2 and IFN-γ production

**Fig. 6:**
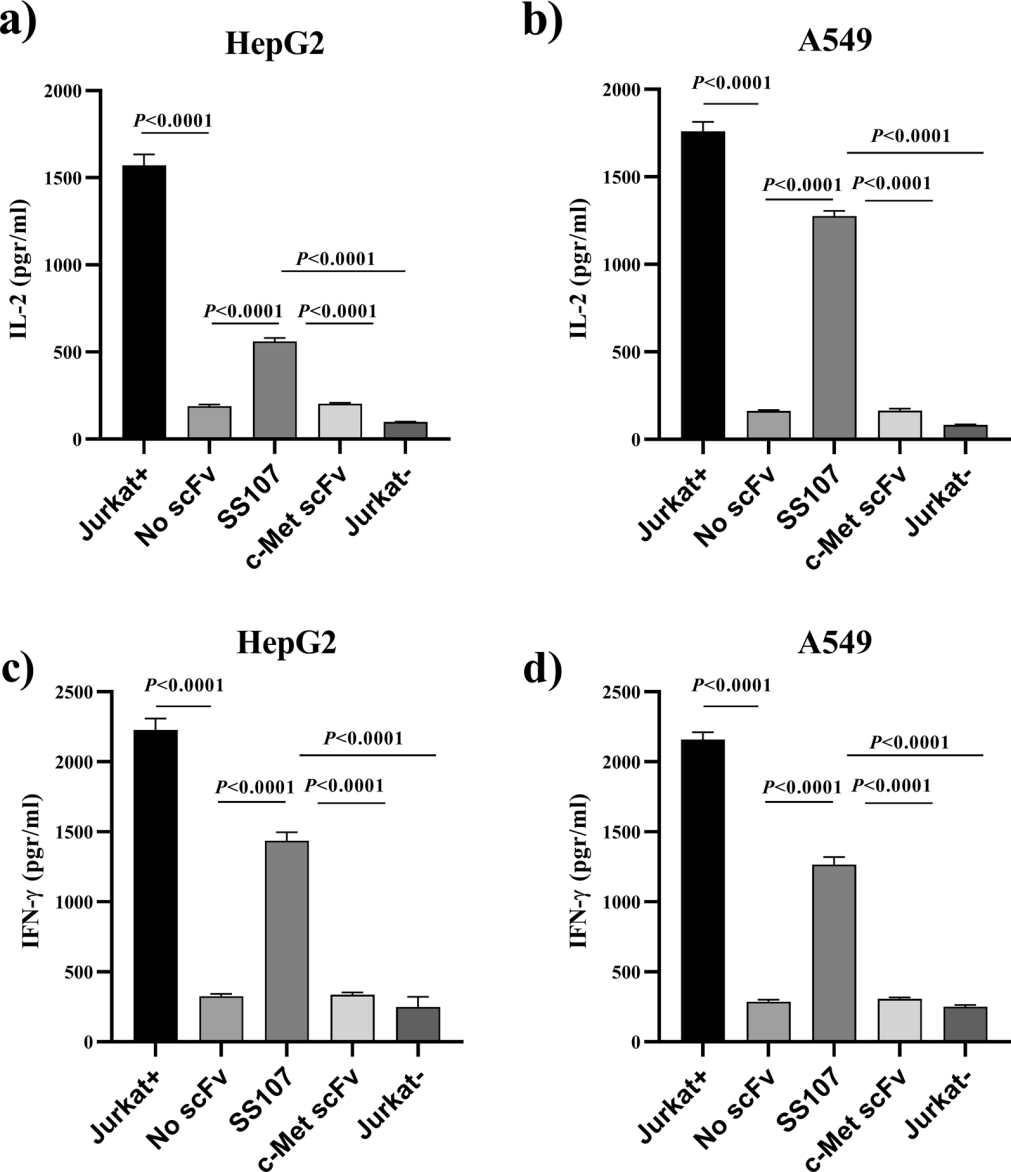
Restoration of IL-2 (a & b) and IFN-γ (c& d) production by addition of anti-PD-1 SS107 scFvto co-cultures of Jurkat cells with HepG2 and A549 cells (*P* < 0.0001). Stimulated Jurkat T cells (Jurkat+) without co-culture and without SS107 treatment served as the positive control. Stimulated Jurkat cells co-cultured with HepG2 and A549 cells treated with an irrelevant scFv (anti-c-Met) and unstimulated Jurkat cells treated with SS107 scFv were used as the negative controls. The results are shown as mean [± SD] amounts of cytokine secreted by the treated cells. Each experiment was performed in triplicate

To test whether the anti-PD-1 scFv can restore the production of IL-2 and IFN-γ by Jurkat T cells, the stimulated Jurkat cells were co-cultured with A549 and HepG2 tumor cells lines, which were pre-treated with IFN-γ to express PD-L1 receptor. Then, SS107 and control scFvs were added to the co-culture systems. After 24 h, the secretion of IFN-γ and IL-2 was measured through ELISA. Co-culturing Jurkat T cells with A549 and HepG2 resulted in a significant decrease in IFN-γ and IL-2 production by the Jurkat cells (Fig. 6). The results demonstrated that the addition of anti-PD-1 blocking scFv, SS107, restored IL-2 produced by Jurkat cells co-cultured with A549 and HepG2 cells by 70 and 57%, respectively. Moreover, the levels of IFN-γ were significantly increased after adding SS107 into A549 and HepG2 cells co-cultured with Jurkat T cells (*P* < 0.0001). There were no significant changes in IL-2 and IFN-γ production when a control scFv (anti-c-Met scFv) was added to the co-culture groups (Fig. 6).

## Discussion

Immunotherapy has emerged as an innovative treatment for several decades, aiming to activate the immune system to recognize and attack cancer cells. Cancer immunotherapy strategies include immune checkpoint inhibitors, cancer vaccines, monoclonal antibodies, adoptive cell therapy (ACT), oncolytic virus therapy, and cytokines, all of which aim to increase the efficacy of treatment [29–31].

Using cancer immunotherapy approaches, especially mAbs, significant success has been achieved in clinical applications in recent years [32]. Having developed in recent years, antibody drugs have decreased adverse effects due to their high specificity, suppressing metastatic tumors, and improving patient survival [33]. Thus, therapeutic antibodies constitute the most rapidly advancing class of new drugs in the pharmaceutical industry [34]. Antibody isolation using the hybridoma technology or transgenic mice needs long-term immunization procedures and screening [32]. Phage display is a technology to select recombinant antibodies like scFvs that bind specifically to various target molecules such as proteins, peptides, cell-surface glycans, and receptors with high affinity [23]. Until now, 14 phage display-derived therapeutic antibodies have been approved by FDA and/or EMA that the first one was against anti-tumor necrosis factor α (TNFα), named adalimumab (Humira) [35].

Phage display technology can make a direct link between antibody phenotype and its encoded genotype in a single-phage particle. Due to in vitro selection of scFvs through phage display technique, specific scFvs can be isolated against poorly immunogenic antigens, such as antigens showing high homology to host animals’ proteins or those that are toxic to their hosts [26, 32]*.* The smaller size of scFvs compared to mAbs (~ 25 vs. 150 kDa) leads to increased penetration ability of scFvs to tumor cells [29, 36]. The absence of an Fc domain in scFvs can decrease the chance of non-specific binding and the resulting undesirable immunogenic responses [37]*.* With all the characteristics mentioned, scFvs can be regarded as promising clinical tools in a wide variety of applications. Also, studies show that other formats of small fragment antibodies (e.g., VL, VH, Fab, and nanobody) can be as efficient as scFvs antibodies. For instance, Fouladi et al*.* successfully isolated a fully human VL antibody fragment with high affinity against the Flap region of urease enzyme of Helicobacter pylori which can be used for H. pylori targeting [38].

More recent attention to immunotherapy studies has focused on immune checkpoint blockade therapies [31]*.* Immune checkpoint receptors are membrane molecules mainly expressed on T, and NK cells whose interactions with their ligands maintain self-tolerance and prevent autoimmunity. Tumor cells can upregulate the surface expression of these receptors in order to induce local immune suppression and inhibit anti-tumor immune response. Cytotoxic T-lymphocytes-associated protein-4 (CTLA4) and PD-1 are two important immune checkpoints that have been well characterized in the field of immuno-oncology [31, 32].

Immune checkpoint inhibition therapies, especially those employing monoclonal antibodies, have shown significant and durable responses in numerous malignancies [29, 39]. Several anti-PD-1 and anti- PD-L1 antibodies have been developed for immunotherapy of melanoma, lung, bladder, skin, and uterus cancers. Clinical results demonstrated that anti-PD-1 mAbs were more successful than anti-CTLA4 mAbs in patients with advanced-stage melanoma [40]. Also, several clinical trials are currently employing anti-PD-1 mAbs in the combination of other antibodies (anti-PD-L1, anti-CTLA4, anti-TIM-3, anti-LAG-3, and anti-TGF-β1 mAbs) in various solid tumor models to overcome the resistance to anti-checkpoint immunotherapies [41]. The superior results of checkpoint inhibitors in clinical trials led to FDA approval of anti-CTLA-4 (Ipilimumab), anti-PD-1 (Nivolumab, Pembrolizumab, and Cemiplimab), and anti-PD-L1 (Atezolizumab, Durvalumab, and Avelumab) mAbs [42].

We reported here the detailed isolation and characterization of specific scFvs against PD-1, an immune checkpoint protein, from a fully human phage display semi-synthetic library. The anti-PD-1 scFvs could successfully bind to PD-1 protein in several ELISA experiments, including polyclonal, monoclonal, and soluble ELISA. Consistent with the data observed in the specificity ELISA test, SS107 scFv did not have any cross-reactivity with other proteins and peptides antigens. Also, cell binding assay through flow cytometry confirmed the antigen-specific binding of the selected anti-PD-1 scFv. We assessed the anti-PD-1 scFv function to restore the production of IL-2 and IFN-γ by Jurkat T cells. In this designed co-culture system, IFN-γ—pre-treated A549 and HepG2 cell lines were employed to express PD-L1. The results revealed that the addition of SS107scFv to the co-culture system could disrupt the interaction of PD-1, expressed on stimulated Jurkat T cells, and PD-L1 and led to restoring IL-2 and IFN-γ production. Specifically, there was no significant release of IFN-γ and IL-2 cytokines from unstimulated Jurkat T cells after incubation with anti-PD-1scFv or from stimulated Jurkat T cells without incubation with the selected scFv. Moreover, when stimulated Jurkat T cells were incubated with an irrelevant anti-c-Met scFv, the secretion of IFN-γ and IL-2 could not be restored. These results revealed that the anti-PD-1 scFvs could enhance T cell reactivity and potently increase cytokine release.

In a similar study, the anti-PD-1 monoclonal antibody Nivolumab was fully characterized. This antibody could bind specifically to PD-1 and, more importantly, could enhance anti-tumor responses and T cell cytokine production [43]. Also, in several studies, anti-PD-1 scFvs have been employed as tumor-targeted therapy tools that resulted in the induction of desirable anti-tumor immune responses. Passaro et al. generated an scFv antibody against PD-1 and engineered a novel oncolytic herpes simplex virus (oHSV) expressing this scFv (NG34scFvPD-1). They reported that in two pre-clinical mouse models of glioblastoma, intratumoral administration of NG34scFvPD-1 could induce both durable and memory anti-tumor responses [44]*.* In another study by Lin et al., an immunotherapeutic herpes simplex virus (HSV) expressing aMPD-1 scFv was developed for local delivery of anti-PD-1 scFv in the tumor microenvironment which contributed to improving antigen cross-presentation in dendritic cells and enhancing anti-tumor T cell activity [45]. These findings provide a rationale for combining checkpoint blockade immunotherapy, like PD-1-scFv, with oncolytic viruses (OVs) for maximizing therapeutic efficacy.

Despite considerable clinical benefits of immune checkpoint blockade therapy, systemic administration of immune checkpoint monoclonal antibodies is associated with immune-related adverse events (IRAEs) [46]. The development of engineered delivery systems is an effective strategy to minimize and control IRAEs. In this regard, chimeric antigen receptor-engineered T (CAR-T) cells have been engineered to secrete PD-1-blocking scFvs. Most recent pre-clinical studies have shown that anti-PD-1 scFv-producing CAR-T cells reduced the related toxicities and improved the safety of immune checkpoint inhibition treatment since the secreted scFvs remained locally in tumor tissues and also increased anti-tumor activity of these engineered CAR-T cells in comparison with the conventional ones [46–48]. Therefore, employing CAR-T cells in combination with anti-PD-1 scFvs could be an effective immunotherapeutic strategy against solid tumors.

## Conclusions

Taken together, the scFv antibodies against immune checkpoint proteins in particular PD-1 are promising tools for cancer combination therapy, and it is anticipated that it will lead to many innovative and high potential constructs for cancer immunotherapy against solid tumors. In our study, we developed a novel human anti-PD-1 scFv, which appeared to possess in vitro properties well-suited for targeting PD-1 protein. However, further investigations should be conducted to evaluate other in vitro and in vivo anti-tumor properties of this scFv.

## Methods

### Reagents

The human semi-synthetic Tomlinson I + J phagemid libraries of scFv fragments cloned in ampicillin-resistant pIT2 phagemid vector were obtained from Cambridge, UK. The human recombinant PD-1 (CD279) antigen was obtained from R&D Systems (Minneapolis, MN, USA), resuspended in PBS (100 μg/ml), and stored at − 20 °C. M13KO7 helper phage was purchased from Source BioScience (Nottingham, UK). Anti-c-Myc and anti-M13 mAbs conjugated to horseradish peroxidase (HRP) were obtained from Roche (Mannheim, Germany). *E. coli* strains TG1 and Rosetta-gami2 were bought from Novagen (Madison, WI, USA). The DNA ladders were purchased from Takara (Dalian, China). DAB (3, 3′-diaminobenzidine), BSA, kanamycin sulfate, ampicillin, phenylmethylsulfonyl fluoride (PMSF) and isopropyl β-Dthiogalactopyranoside (IPTG) were obtained from Sigma-Aldrich Co. (St Louis, MO, USA). FITC-labeled anti-M13 bacteriophage G8p capsid antibody and mouse IgG isotype control (FITC-conjugated) were purchased from antibodies-online (GmbH, USA). Mouse anti-human PD-1 (clone J116) and FITC-conjugated rat anti-mouse IgG were bought from eBioscience. A purified anti-human CD274 (PD-L1) antibody was obtained from Biolegend (San Diego, CA, USA). HepG2 and A549 cells were cultured in complete DMEM, and Jurkat cells were cultured in RPMI 1640. All cell lines were provided by the National Cell Bank of Iran (Pasteur Institute of Iran, Tehran, Iran). Recombinant human IL-2 and INF-γ were purchased from R&D Systems. All other reagents were obtained from Sigma-Aldrich (St Louis, MO, USA).

### Library amplification

To rescue scFv-displaying phages, 500 μl of phagemid Tomlinson I + J library was added to 200 ml of 2XYT supplemented with ampicillin (120 μg/ml) and incubated at 37 °C with shaking at 250 RPM until the OD_600_ of 0.4 was achieved. Then, for each milliliter of bacterial culture, about 5 × 10^11^ CFU of helper phage M13K07 was added and incubated at 37 °C for 1 h. Next, the bacteria were collected by centrifugation for 10 min at 4000 rpm at 4 °C, resuspended in 100 ml of fresh 2XYT containing 120 μg/ml ampicillin and 50 μg/ml kanamycin, and incubated with shaking at 30 °C overnight. Then, the rescued phages were precipitated from the supernatant by adding 20% (w/v) polyethylene glycol 6000/2.5 M NaCl (PEG/NaCl) and incubated on ice for 1 h. After centrifuging for 20 min at 14,000 rpm at 4 °C, the phages were resuspended in 4% MPBS (skimmed milk in PBS) [49].

### Biopanning process

The amplified Tomlinson I + J library was used for selecting high-affinity phage clones expressing anti-PD-1 scFvs. Four sequential rounds of panning were performed on recombinant human PD-1 protein in 24-well plates. In this project, a subtractive screening of biopanning was used in which one well was coated with recombinant human PD-1 as the target antigen and another with BSA as an irrelevant antigen. A 24-well plate was coated with PD-1 (1 μg/ml) and BSA (1 μg/ml) as a subtraction well in sodium bicarbonate coating buffer (pH 9.6) and incubated overnight at 4 °C. The wells were then washed three times with PBS and blocked with 5% MPBS at 37 °C for 90 min with mild shaking conditions. The amplified phages (10^12^ CFU/ml in 5% MPBS) were added to the BSA coated well and incubated for 1 h (first 30 min with shaking and later 30 min without shaking) at 37 °C. The unbound phages were transferred to the target well coated with PD-1 and incubated for 1 h at 37 °C. Afterward, the supernatant was discarded, and unbound phages were removed by washing ten times with PBST (PBS containing 0.5% Tween 20), five times with PBS, and lastly, with distilled water. The PD-1-binding phages were eluted from the well with 1 ml of 100 mM triethylamine (TEA) for 10 min at room temperature (RT) and then neutralized with 1 ml of 1 M Tris–HCl buffer (pH 7.5) (output1). For phage amplification, after each round of panning, 100 μl of eluted phage was utilized to infect 5 ml of competent *E. coli* TG1 (OD_600_ = 0.5) for 1 h at 37 °C. Then, ampicillin was added to the final concentration of 120 μg/ml, and the transformed bacteria were incubated at 37 °C for an additional 1 h. Subsequently, M13KO7 helper phage was added to the enriched phagemids and incubated by shaking at 37 °C for 1 h. The kanamycin was added to a final concentration of 50 μg/ml and incubation continued for 1 h. Finally, the bacteria were added to 20 ml of 2XYT medium containing 120 μg/ml ampicillin and 50 μg/ml kanamycin and grown with agitation at 37 °C for 16–20 h. Afterward, the process of phage purification was performed as described above, and the panning process was rehashed for another three rounds. The stringency of the panning conditions in each round was increased in two ways. First, the Tween-20 concentration was increased from 0.5 to 1%, 2%, and 4% for rounds 1, 2, 3, and 4 of panning, respectively. Second, the washing steps were increased from 10 to 15, 20, and 25 times in the panning process for more stringent selection [50].

### Polyclonal phage ELISA

The purified anti-PD-1 output phages from four rounds of panning were screened by polyclonal phage ELISA to determine the binding activity of phage scFvs. The wells of MaxiSorp 96-well plates were coated with 100 μl of 1 μg/ml PD-1 and BSA as the negative control and then incubated overnight at 4 °C. After twice washing with PBS, the wells were blocked with 250 μl of 5% MPBS for 90 min with mild shaking at 37 °C. Afterward, 10^12^ CFU/ml of amplified output phages were added to the wells and incubated at 37 °C for 1 h. The wells were washed four times with 0.05% PBST for the elimination of nonspecific phages. Then, a 5000-fold diluted solution of murine anti-M13-HRP was added to the wells, incubated at 37 °C for 1 h, and then washed three times with PBST and one time with PBS, respectively. Following that, the color was established using 100 μl/well of tetramethylbenzidine (TMB) substrate and the plate was incubated in the dark at RT for up to 30 min. Finally, the enzyme reaction was stopped by adding 1 M sulfuric acid. The absorbance was measured at 450 nm using an ELISA plate reader (BP-800; Biohit Inc., Neptune, NJ, USA) [51].

### Screening of PD-1-specific clones by monoclonal phage ELISA

Following polyclonal phage ELISA, 110 individual clones from output phages were randomly selected to determine the specificity of PD-1 binding by monoclonal phage ELISA. Briefly, *E. coli* TG1 cells (OD_600_ = 0.5) were infected with 100 μl of each output phages of panning, incubated for 1 h at 37 °C, and then spread on LB agar plates containing ampicillin(120 μg/ml) and incubated for overnight at 37 °C. Subsequently, single colonies were removed from the plates and cultured in an ampicillin-containing LB culture medium. Afterward, the phages were produced by the addition of M13K07 helper phages. Wells of MaxiSorp 96-well plates were coated with PD-1 and BSA (as the negative control). The screening procedure of antigen-specific clones was performed as described above for polyclonal phage ELISA [52].

### Expression of soluble PD-1 scFvs

For the soluble production of anti-PD-1 scFvs, the strongest positive phages in monoclonal phage ELISA were used to infect *E. coli* Rosetta-Gami 2 (a non-suppressor strain), and transformants were cultured in 20 ml 2XYT medium containing 120 μg/ml ampicillin while shaking at 37 °C until the OD_600_ of 0.9 was attained. The scFv expression was induced with 1 mM IPTG and incubated for 18 h at 30 °C. Then, bacterial cells were centrifuged at 3700 rpm for 20 min and resuspended first in 1mMPMSF and second in TES (Tris–EDTA–Sucrose) buffer for osmotic shock process and incubated on ice for 1 h. Afterward, TES/4 buffer (TES diluted 1:4 in water) was added and incubated on ice for 2 h with mild shaking. Then, they were centrifuged for 30 min at 10,000 rpm and the supernatants, including the periplasmic scFv proteins, were collected. To test the binding activity of each produced scFv, soluble ELISA was performed with an anti-c-Myc antibody conjugated to HRP (1: 2000 dilution) as the secondary antibody [53].

### PCR and sequencing

Individual clones that were positive in soluble phage ELISA were checked for the existence of full-length VH and VL inserts by PCR (polymerase chain reaction). The scFvs genes were amplified with primer pairs LMB3 (5′ CAGGAAACAGCTATGAC 3′), and pHEN (5′CTATGCGGCCCCATTCA 3′) and the PCR products were run on 1% agarose gel containing Green viewer in TAE (Tris–acetate-EDTA) buffer. Furthermore, scFv genes were purified with the QIAquick Gel Extraction Kit (Qiagen GmbH, Hilden, Germany) and sequenced by Bioneer Corporation (Daejeon, South Korea).

### Specificity of the soluble scFv

The ELISA experiment was performed to assess the possible cross-reactivity of isolated scFvs with a variety of antigens. PD-1, 4-1BB, IGF-1R, c-Met, Fzd7, ROR1, skimmed milk, and BSA antigens were individually coated onto 96-well ELISA plates at 1 μg/ml and incubated overnight at 4 °C. The wells were blocked and then incubated with selected scFvs. The reaction between coated antigens and anti-PD-1scFvs was detected by HRP conjugated anti-c-Myc antibody.

### Western blot analysis

In order to confirm the production of soluble scFv proteins, western blot analysis was performed. After periplasmic expression and purification of soluble anti-PD-1 scFv antibodies, they were quantified through Bradford assay, and equal amounts of protein (1 µg) per sample were loaded on a 10% discontinuous sodium dodecyl sulfate–polyacrylamide gel electrophoresis (SDS–PAGE). Subsequently, the gel was transferred onto nitrocellulose membranes using a semidry transfer device (Amersham Biosciences, Freiburg, Germany). The membrane was blocked with the blocking solution (3% BSA) at 4 °C overnight and incubated in a solution of PBS containing a 1:2000 dilution of HRP conjugated anti-c-Myc for 2 h at 37 °C with gentle agitation. After washing six times with PBS-Tween 0.1% buffer, the bound antibody was revealed by staining with DAB.

### Cell binding analysis by flow cytometry

The cell binding assay was done to determine the cell surface binding ability of the selected scFv antibodies. The Jurkat T cells were cultured in RPMI 1640 medium supplemented with 10% fetal bovine serum (FBS), 100 μg/ml streptomycin, and 100 U/ml penicillin at 37 °C in a humidified CO2 incubator. For Jurkat T cell stimulation, the cells were treated with 50 ng/ml PMA (phorbol myristate acetate) and 100 ng/ml ionomycin and incubated for 96 h. To sum up, 4 × 10^5^ stimulated Jurkat cells were treated with the selected phage-displayed scFv (10^12^ CFU/ml) and incubated for 45 min at 4 °C. After that, the cells were washed three times with washing buffer (PBS-5%FBS) and treated with FITC-conjugated anti-M-13 antibody for 45 min at 4 °C. Concurrently, the cells were also stained with a commercial mouse anti-PD-1 mAbas a positive control and an anti-c-Met scFv as a negative control [53]. The amount of scFvs bound to the PD-1 receptor was measured by a PartecPASIII (Partec GmbH, Munster, Germany) flow cytometer. The FlowJo 7.6.1 software was employed for analyzing the stained cells (Tree Star, Inc., Ashland, OR, USA).

### Cytokine analysis by ELISA

Human non-small cell lung cancer line A549 and human hepatocellular carcinoma line HepG2 were cultured in the DMEM medium at 37 °C in a humidified CO2 incubator and grown to 80% confluency. These cell lines were pre-treated with IFN-γ (500 unit/ml) to increase PD-L1 expression and incubated for 24 h. Then, the cells were co-cultured with stimulated Jurkat T cells (under optimal stimulation conditions as described above) at a 2:1 effector-to-target (E-T) ratio with or without selected the scFv for 24 h. Supernatants were collected and employed for IL-2 and IFN-γ assay through ELISA.

## Supplementary Information

**Additional file 1: Figure S1**. The original gel and blot image of Fig. 4. Expression of soluble scFv fragment in Rosetta-Gami 2 was evaluated by SDS-PAGE (a) and western blot analysis (b). M: protein marker; Lane 1: total lysate from non-induced *E. coli* Rosetta-Gami 2 as negative control; Lane 2: SS107 scFv; The molecular weight of SS107 scFv was about 28 kDa.

## Data Availability

All authors declare that the data generated or analyzed during this study are included in this published article and its Additional file 1.

## Abbreviations

PD-1: Programmed death 1
ITIM: Iimmuno-receptor tyrosine-based inhibitory motif
ITSM: Immuno-receptor tyrosine-based switch motif
mAb: Monoclonal antibody
Fab: Fragment antigen-binding
scFv: Single-chain variable fragment
VL: Variable light
VH: Variable heavy
ELISA: Enzyme-linked immunosorbent assay
*E. coli*: *Escherichia coli*
IPTG: Isopropyl β-D-thiogalactopyranoside
HRP: Horseradish Peroxidase
DAB: Diaminobenzidine
BSA: Bovine serum albumin
PMSF: Phenylmethylsulfonyl fluoride
PBS: Phosphate buffered saline
MPBS: Skimmed milk in PBS
CFU: Colony-forming unit
PEG: Polyethylene glycol
TEA: Triethylamine
TMB: Tetramethylbenzidine
TES: Tris–EDTA–sucrose
SDS–PAGE: Sodium dodecyl sulfate–polyacrylamide gel electrophoresis
FBS: Fetal bovine serum
PMA: Phorbolmyristate acetate
OD: Optical density
IGF-1R: Insulin-like growth factor 1 receptor
HGFR: Hepatocyte growth factor receptor
ROR1: Receptor tyrosine kinase like orphan receptor 1
Fzd7: Frizzled-receptor 7
ACT: Adoptive cell therapy
TNFα: Tumor necrosis factor α
CTLA4: Cytotoxic T-lymphocytes-associated protein-4
oHSV: Oncolytic herpes simplex virus
OV: Oncolytic viruses
IRAEs: Immune related adverse events
CAR-T: Chimeric antigen receptor-T
RT: Room temperature
PCR: Polymerase chain reaction
TAE: Tris-acetate-EDTA
OV: Oncolytic viruses
