# Supplementary material for: Development of a human phage display-derived anti-PD-1 scFv antibody: an attractive tool for immune checkpoint therapy
Source: BMC Biotechnol. 2022 Aug 23;22:22. doi: 10.1186/s12896-022-00752-8 (PMC9396865; doi:10.1186/s12896-022-00752-8)
Supplement: Supplementary file 1 — Additional file 1: Figure S1. The original gel and blot image of Fig. 4. Expression of soluble scFv fragment in Rosetta-Gami 2 was evaluated by SDS-PAGE (a) and western blot analysis (b). M: protein marker; Lane 1: total lysate from non-induced E. coli Rosetta-Gami 2 as negative control; Lane 2: SS107 scFv; The molecular weight of SS107 scFv was about 28 kDa. [file 12896_2022_752_MOESM1_ESM.docx]

**Development of a human phage display-derived anti-PD-1 scFv antibody: an attractive tool for immune checkpoint therapy**

Sepideh Safaei Ghaderi, Farhad Riazi-Rad, Elmira Safaie Qamsari, Salman Bagheri, Fatemeh Rahimi-Jamnani, Zahra Sharifzadeh

**Original picture of Fig 4**

1. **(b)**

**M 1 2 M 1 2**


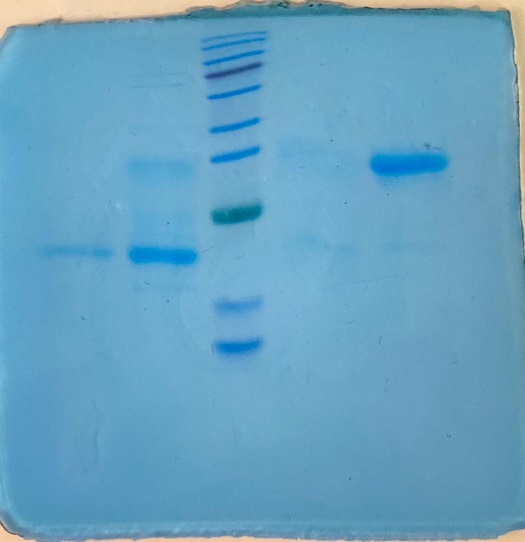

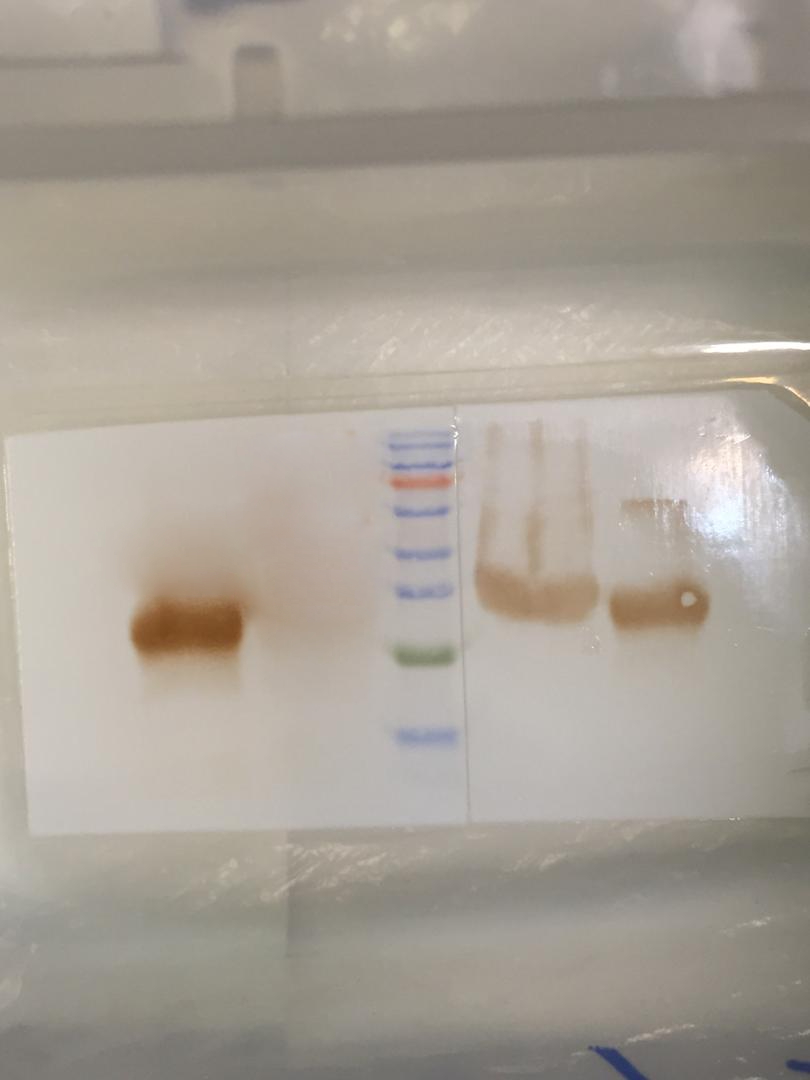


**Figure S1.** Expression of soluble scFv fragment in Rosetta-Gami 2 was evaluated by SDS-PAGE (a) and western blot analysis (b). M: protein marker; Lane 1: total lysate from non-induced E. coli Rosetta-Gami 2 as negative control; Lane 2: SS107 scFv; The molecular weight of SS107 scFv was about 28 kDa.
